# Validation study of the Spanish version of the Last-7-d Sedentary Time Questionnaire (SIT-Q-7d-Sp) in young adults

**DOI:** 10.1371/journal.pone.0217362

**Published:** 2019-05-29

**Authors:** Mireia Felez-Nobrega, Judit Bort-Roig, Kieran P. Dowd, Katrien Wijndaele, Anna Puig-Ribera

**Affiliations:** 1 Sport and Physical Activity Research Group and Centre for Health and Social Care Research, Department of Physical Activity and Sports Sciences, University of Vic - Central University of Catalonia, Vic, Barcelona, Spain; 2 Department of Sport and Health Sciences, Athlone Institute of Technology, Athlone, Westmeath, Ireland; 3 MRC Epidemiology Unit, School of Clinical Medicine, University of Cambridge, Cambridge, United Kingdom; Universidad Europea de Madrid, SPAIN

## Abstract

There are few valid instruments to assess domain-specific sedentary behaviours (SB) among Spanish-speaking populations. This study validated the original English version of the last 7 days SB questionnaire (SIT-Q-7d) into Spanish (Castilian). A total of 151 undergraduates (52% male, 21.19±2.57 yrs.) wore an activPAL^3M^ (AP^3M^) for 7 days and subsequently completed the Spanish version of the SIT-Q-7d (SIT-Q-7d-Sp). A subsample of 30 participants (70% male, 22.89±1.54 yrs.) simultaneously wore the AP^3M^ and used a domain-log to register the context where the SB occurred. The SIT-Q-7d-Sp differed significantly from the AP^3M^, overestimating sitting time by an average of 60.69 mins.d^-1^ (all p<0.016). No significant differences were observed between the two measures for weekend total sitting time. The SIT-Q-7d-Sp did not differ significantly from the AP^3M^ +Log for meal, work, and transportation-based sitting time (all p>0.016). However, screen-based and other leisure-based sitting activities were significantly overestimated (ranging from 94.68 mins.d^-1^ to 234.08 mins.d^-1^, p<0.001). The SIT-Q-7d-Sp appears to provide acceptable estimates of sitting time during transportation, occupational and meal-based domains. The SIT-Q-7d-Sp is not an appropriate measure of SB when examining total sitting time and leisure-based SB in young adults. For total sitting time and leisure-based SB, the use of objective measures is recommended.

## Introduction

Sedentary behaviour (SB; i.e. any waking activity characterized by an energy expenditure of ≤1.5 METs (Metabolic Equivalent of Tasks) while in a sitting, reclining or lying posture; [1] is a complex phenomenon that occurs in different domains (occupational, transportation, leisure and domestic), dimensions (i.e. duration, frequency) and modes (e.g. TV, computer use, reading) [2]. Evidence has highlighted that higher levels of SB are related to higher all-cause mortality [3,4]; increased risk of chronic diseases [4], poorer mental well-being [5] and increased risk of depression [3,4,6]. Importantly, some modes of SB, and more specifically television-viewing time, may be more strongly related to health than others [4,7–9]. Given that targeting different domains and modes of SB could improve health, developing domain-specific SB measurement tools with good measurement properties has become a critical issue.

SB assessment methodologies in free-living conditions include both subjective (i.e. self-report) and objective measurements (i.e. wearable monitors) [10]. While objective monitors provide more accurate assessments in terms of duration and frequency (i.e. SB total time, number and length of SB bouts [11]), these are costly and currently less able to distinguish between different domains or modes of SB (10).

Self-reported tools for SB (i.e. recall questionnaires) are susceptible to random and systematic reporting errors, including social desirability bias [10,12]; however, these can be implemented on a large scale, are relatively inexpensive and easy to administer [13]. Importantly, recall questionnaires can provide additional information about the domain where the behaviour takes place [13,14].

Many questionnaires are currently available for the measurement of SB in adult populations. A recent review identified 35 adult questionnaires which have undergone psychometric testing [14]. SB questionnaires assess global measures of sitting time via a single item question of total daily sitting time (i.e. International Physical Activity Questionnaire–IPAQ, [15]) multiple SB that occur in different domains (i.e. Marshall Sitting Questionnaire–MSQ, [16]; Sedentary Behaviour Questionnaire–SBQ, [17]; and Past-day Adults’ Sedentary Time–PAST, [18]) or work-place sitting time (i.e. Occupational Sitting and Physical Activity Questionnaire–OSPAQ; [19]). One of the most comprehensive self-reported questionnaires is the Last 7-day Sedentary Time Questionnaire (SIT-Q-7d), [20]. This tool was designed to assess volume and patterns of sedentary time in adults aged 20–60 years. The questionnaire assesses total and domain-specific SB with a reference frame of the last 7 days. It provides estimations for weekday and weekend−specific sitting time (minutes/day) across five different domains: meals, transportation, occupation, leisure screen time and time spent sedentary in other activities (e.g. sitting while reading, performing household tasks, providing care for relatives, performing hobbies, socializing or listening to music).

While most available self-reported tools have been developed and validated for English-speaking populations, Spanish versions of SB questionnaires and studies examining their psychometric properties are scarce [14]. Few studies have examined validity of self-reported sitting time tools in the Spanish population, focusing on single non-domain specific items [21], patients with chronic conditions such as fibromyalgia [22], or adolescents [23]. Other interventional studies have been merely back-translating English original SB questionnaires into Spanish, without assessing their psychometric properties [24–26].

As Spanish is the second most spoken language in the world, with nearly 500 million native speakers, developing an accurate measure of SB for Spanish-speaking populations is of public health significance. Given that the SIT-Q-7d appears to have satisfying qualities for assessing SB in adults, and the scarcity of available and validated questionnaires of SB in Spanish, we aim to conduct a validation study of the SIT-Q-7d-Sp in a subsample of the Peninsular-Spanish population.

## Materials and methods

### Participants

This analysis was completed within a previously collected dataset of a larger project [27,28]. A total of 163 undergraduate students from the University of Vic-Central University of Catalonia (UVIC-UCC; Northeastern region of Spain) participated in the study. Inclusion criteria involved being a native Spanish speaker and aged between 18–25 years old. Ethical approval was obtained by the research ethics committee of UVIC-UCC (Comité de ética de investigación clínica de la Fundación Osona para la Investigación y la Educación Sanitarias). All participants provided written informed consent prior to participation.

### Procedures

The original SIT-Q-7d questionnaire and recommended processing codes are provided in http://www.mrc-epid.cam.ac.uk/research/resources/. A linguistic and cultural adaptation of the SIT-Q-7d for the Peninsular-Spanish speaking population was performed following Hambleton `[29] guidelines and recommendations. First, two Spanish native-speaking translators independently translated the original English version of the SIT-Q-7d into Spanish. The researcher and translators compared both Spanish versions in order to create a first draft of the Spanish version. Then, an English native-speaking translator back translated this first Spanish draft into English. All translators were experts in translating health instruments and native speakers of the target languages. Finally, all translators and the researcher reviewed and discussed the back-translation to identify any discrepancies between the meaning of the translation and the original questionnaire. Based on the comparison between the original English version and the version that was back translated to Spanish, the last modifications were conducted on the Spanish questionnaire, yielding the final Spanish version of the SIT-Q-7d-Sp (see S1 Appendix).

Subsequently, criterion validity was evaluated against the activPAL^3M^ (AP^3M^) for total sitting time. Participants were instructed to wear the device for 24h hours per day during a 7-day period. After the 7 days wearing the device, participants completed the SIT-Q-7d- Sp questionnaire. For domain-specific sedentary time, a subsample of participants (n = 30) simultaneously wore the AP^3M^ and registered in a domain-log the context where the sedentary activities occurred, which was handed in before completion of the SIT-Q-7d-Sp (see S2 Appendix).

### Measurements

The SIT-Q-7d-Sp assessed SB minutes per day (mins·d^-1^) across different domains during weekdays and weekends. The meal domain included time spent for breakfast, lunch and dinner. Response categories ranged from periods of 10-minute blocks and 15-minute blocks, with the last option being >1 hour. The transportation domain included time spent to and from occupation and moving about apart from occupation. Response categories were organized into 15- minute blocks, 30-minute blocks and 1-hour blocks, with the last option being >7 hours. The occupation domain was the sum of time (mins·d^-1^) spent seated while working, studying or volunteering. Leisure screen time was the sum of the amount of time/day seated while watching TV, using a computer apart from work and playing sedentary computer games. Time spent sedentary in other activities was the sum of mins·d^-1^ spent sitting while reading, performing household tasks, providing care for relatives, performing hobbies, socializing, listening to music or other activities. Response categories in occupation, leisure screen time and other activities domains ranged from periods of 15-minute, 30-minute and periods of 1 hour with the last option being 8 hours for the occupation domain and 7 hours for the leisure screen and other activities domain.

The following sections of the original questionnaire were excluded in the Spanish version (see S1 Appendix): “travelling as part of your occupation”, as it created confusion among participants during the pilot testing, “snacking while watching TV” although it could be an important factor associated with SB, this domain was not validated in the original questionnaire and goes beyond SB assessments, and “breaks in sitting time” due to the difficulty of recalling this behaviour for its intermittent nature and the poor reliability and validity shown in the original validation [20].

The SIT-Q-7d scoring system assigns the midpoint values for each response category (e.g. 1–10 minutes = midpoint value 5). For the final response categories such as ‘more than 1 hour,’ the midpoint was calculated by adding half of the difference between the upper and lower cut-points of the previous category (i.e. more than 1 hour = midpoint value 67.5).

The AP^3M^, weighing 9g and measuring 25x45x5mm, was used to quantify total sedentary time during free-living conditions. This device is widely considered the “gold standard” for measuring SB [11,30]. The AP^3M^ was placed in a small flexible nitrile sleeve to waterproof the device and was attached to participants’ right thigh using a transparent film (10 x 10cm of hypoallergenic Tegaderm Foam Adhesive Dressing). Recording began at 12 midnight on the day of AP^3M^ application to avoid the collection of potentially reactive data. The devices were programmed to stop recording at 12 midnight of day eight, which ensured the inclusion of seven completed days of 24 hour recordings. Participants were asked to record removal reasons over the 7-day period. Data were initialized using activPAL Professional Software (version 7.2.32) and further processed using Microsoft Excel 2010 (Redmond, WA, USA) and MATLAB (MathWorks, Natick, MA, USA). The protocol used for data collection, reduction and analysis is described in detail elsewhere [31]. Briefly, data were included in the analyses if participants provided a minimum of 4 valid days of recording (including 1 weekend day). Valid days were defined as a day with ≤4h of nonwear time during identified waking hours. Non-wear time was defined as a period with ≥60 minutes of consecutive zero activity counts.

An adapted Spanish version of the log employed in the original study was used (see S2 Appendix). Each day (7 in total) was shown in a different page and days were broken into 15 minutes segments. The domain log included a list of 10 different categories (1 sleep, 2 meal, 3 transportation, 4 work, 5 study, 6 volunteering, 7 TV/smartphone use/DVD, 9 non-work-related computer use/video games, and 10 “other” -e.g. reading, hobbies-). Participants were asked to assign a code from the list to each 15-minute segment, during 24 hours and for 7 days. Only activities that occurred in a sitting, reclining or lying posture were registered. More details about the characteristics of the domain-log can be found elsewhere [20]. Data from the domain-log was merged with the AP^3M^ data to calculate sedentary time for each domain activity (i.e. meals, transport, occupation, leisure screen time, other leisure sedentary activities). The data obtained from the log and AP^3M^ (AP^3M^ +Log) was used as criterion measure to assess domains’ validity of SIT-Q-7d-Sp [20,30,32].

### Statistical analysis

To enable comparison between the findings of the SIT-Q-7d-Sp and the AP^3M^ data, self-reported total sitting time and domain specific sitting time from the SIT-Q-7d-Sp was averaged for weekdays, weekend days and for all 7 days. All variables were examined for normality, and descriptive characteristics ─i.e. mean and standard deviation (SD); median and interquartile range (IQR)─ were presented for total sitting times and domain specific sitting times on weekdays, weekend days and for the previous 7 days.

Associations between SIT-Q-7d-Sp and the AP^3M^ / AP^3M^ +Log were examined using Pearson’s correlation coefficient or the Spearman rank-order correlation coefficients. Paired samples t-tests or Wilcoxon signed-rank tests were used to examine the differences between self-reported and objectively determined total and domain-specific sitting time, depending on normality of the distribution of the estimates involved. Bland-Altman plots were constructed to examine the mean bias and limits of agreement (LoA) for all SIT-Q-7d-Sp variables in comparison with the corresponding AP^3M^ determined sitting variables. A Bonferroni correction was applied to decrease the risk of committing Type 1 errors. The alpha level for statistical significance for the total sitting time validation was set at p<0.016, while an alpha level of p<0.0038 was set for domain specific sitting time. For all other analysis, an alpha level of p<0.05 was set. All statistical analysis was conducted using IBM SPSS Statistics 25 (SPSS, Inc, an IBM Company, Chicago, IL) and Microsoft Excel (Microsoft Corporation, Microsoft Excel 2016, WA, USA).

## Results

### Total sitting time

**Fig 1:**
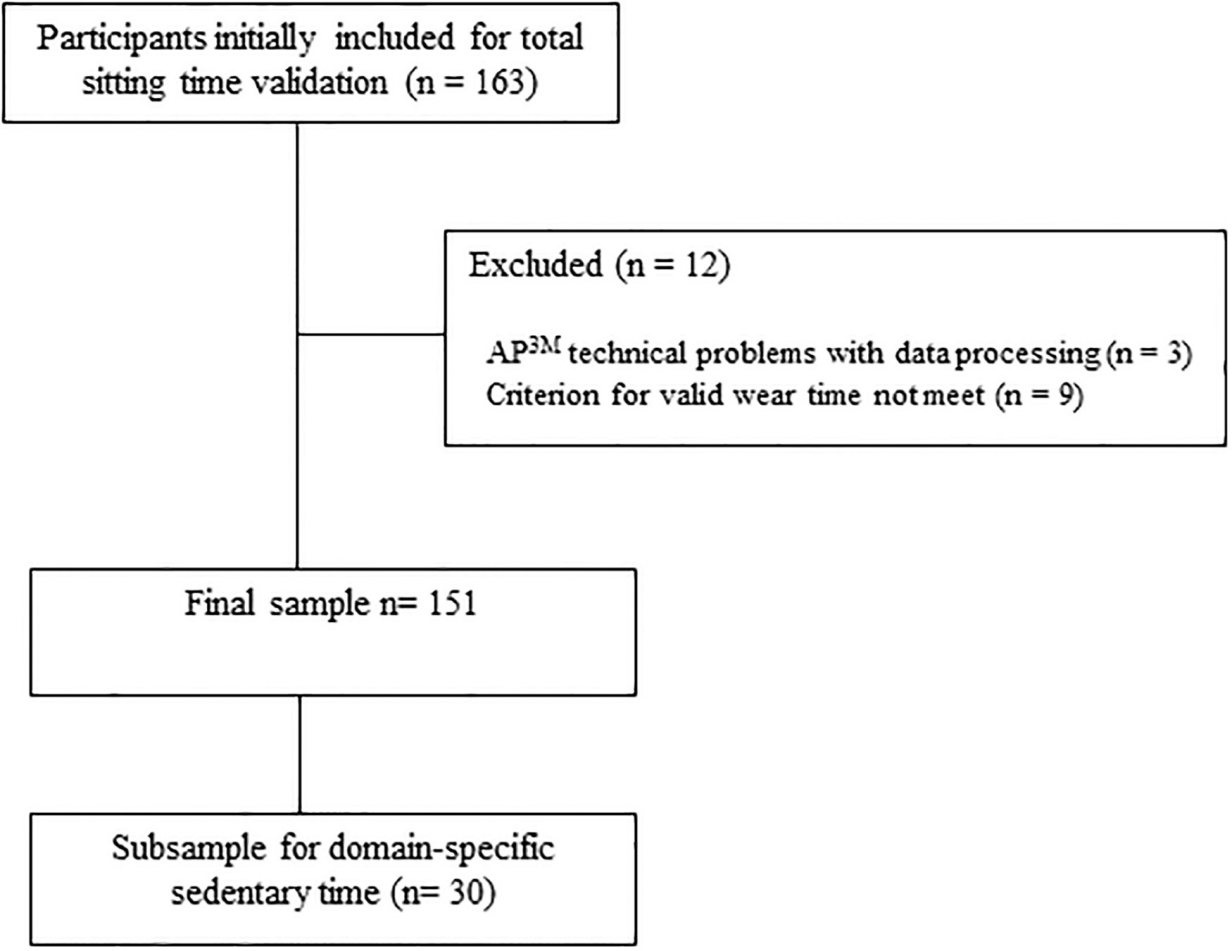
Samples flowchart.

A total of 3 participants were removed from the analysis due to technical problems with data processing, while 9 participants were removed due to the criterion for valid wear time not being met (i.e. at least 3 weekdays and 1 weekend day). After exclusions, a total of 151 participants (78 male, 73 female; 21.19±2.57 yrs.) provided SIT-Q-7d-Sp and AP^3M^ data for this component of the study (Fig 1). Of the 151 included participants, 137 provided 6 valid days of measurement, 12 provided 5 valid days of measurement while 2 provided 4 valid days of measurement.

Descriptive characteristics of the average of total waking mins·d^-1^ spent sitting on weekdays, weekend days and previous 7 days from the SIT-Q-7d-Sp and the AP^3M^ are presented in Table 1. Significant differences between the SIT-Q-7d-Sp and the AP^3M^ were observed for total sitting time on both weekdays (median difference = 32.99; IQR = 296.16; p = 0.01) and total sitting time over the past 7 days (median difference = 38.57; IQR = 258.54; p = 0.01). Furthermore, there was a significant association between the SIT-Q-7d-Sp and the AP^3M^ for total sitting time on weekdays (rho = 0.24; p<0.016) and total sitting time over the past 7 days (rho = 0.28; p<0.001). For weekend total sitting time, results showed no significant association between the two measures (rho = 0.14; p>0.016).

**Table 1:** Descriptive characteristics of the average minutes spent sitting from SIT-Q-7d-Sp and the AP^3M^ [median (interquartile range)], Intraindividual Differences [median (interquartile range)] and results of the Wilcoxon Signed Rank.

N = 151	SIT-Q-7d-Sp	activPAL	Difference	Rho
**Total Sitting Time** **[Weekday (mins·d**^**-1**^**)]**	687.00 (317.00)	639.58 (114.81)	32.99 (296.16)*	0.24*
**Total Sitting Time** **[Weekend Day (mins·d**^**-1**^**)]**	540.00 (285.00)	579.00 (135.65)	2.28 (315.45)	0.14
**Total Sitting Time** **[Average 7 Day (mins·d**^**-1**^**)]**	644.64 (285.71)	609.00 (99.60)	38.57 (258.54)*	0.28**

* P<0.016;

** P<0.001

**Fig 2:**
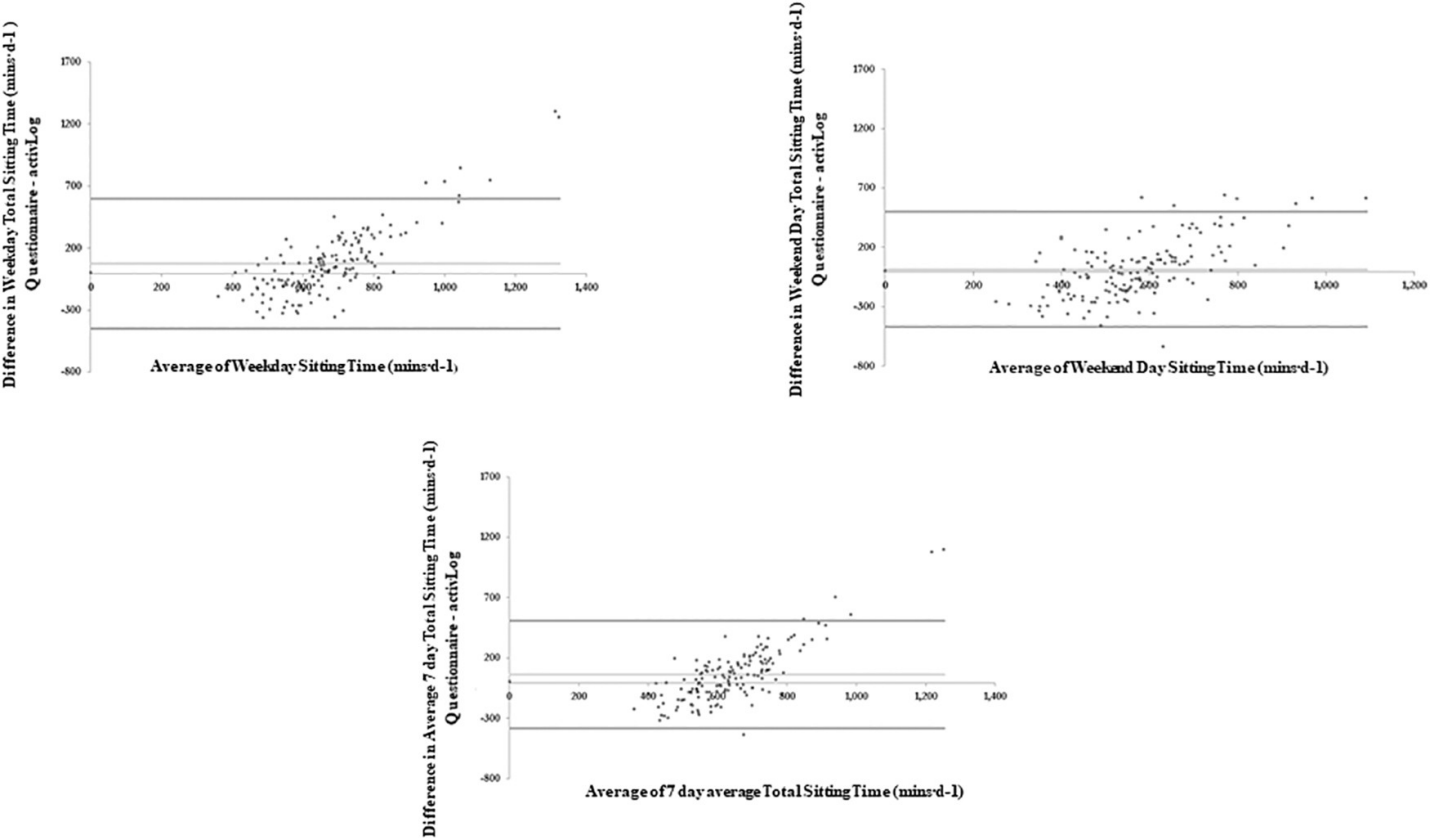
Bland-Altman plots of absolute agreement of total sitting time on weekdays, weekend days and average of the last 7 days derived from the last 7-day Sedentary Time questionnaire with the equivalent variable derived from the AP^3M^.

Bland-Altman plots were used to graphically compare the differences between the two measures (Fig 2 and Table 2). Fig 2 plots the mean sitting time (weekdays, weekend days and sitting time over the past 7 days) measured by the SIT-Q-7d-Sp and the AP^3M^ against the difference of the time spent sitting during each period between the SIT-Q-7d-Sp and the AP^3M^. Participants over-reported their total sitting time on weekdays, with a positive mean bias of +73.46 mins·d^-1^. For total sitting time on weekend days, a substantially smaller positive mean bias of +15.05 mins·d^-1^ was identified, with slightly narrower LoA. Overall, total sitting time over the past 7 days highlighted a positive mean bias of +60.69 mins·d^-1^. A significant positive correlation was observed for the difference between the SIT-Q-7d-Sp and the AP^3M^ for total sitting time on weekdays (rho = 0.72; p<0.001), weekend days (rho = 0.58; p<0.001) and total sitting time over the past 7 days (rho = 0.70; p<0.001). In combination, these findings suggest that those with the highest levels of sitting time over-reported their sitting time to a greater degree than those with lower levels of sitting time on weekdays, weekend days and overall for the past 7 days.

**Table 2:** Mean bias and limits of agreement for total sedentary time on weekdays, weekend days and average 7-days.

	Mean Bias	Upper LoA	Lower LoA
**Total Sitting Time [Weekday (mins·d**^**-1**^**)]**	73.46	598.22	-450.29
**Total Sitting Time [Weekend Day (mins·d**^**-1**^**)]**	15.05	497.85	-467.75
**Total Sitting Time [Average 7 Day (mins·d**^**-1**^**)]**	60.69	506.82	-385.45

LoA: Limits of agreement

### Domain-specific sitting time

A total of 30 participants (21 male, 9 female; 22.89±1.54 yrs.) provided data on domain-specific SB. Two participants were removed from the analysis due to the criterion for valid wear time not being met (i.e. at least 3 weekdays and 1 weekend day), and 6 participants were removed due to incomplete data of the domain log. Of the 30 participants included, 4 provided 7 valid days of measurement, 20 provided 6 valid days of measurement, 4 provided 5 valid days of measurement while 2 participants provided 4 valid days of measurement.

Descriptive characteristics of domain specific sitting variables (sitting time during meal times, sitting time while at work, sitting time for transportation, screen-based sitting time and other leisure-based sitting activities) are presented in Table 3. No significant differences were observed between the SIT-Q-7d-Sp and the AP^3M^ +Log determined sitting time for meal time, work time or transportation on weekdays, weekend days or on the past 7 days. However, significant differences were observed between measures for screen-based sitting time and other leisure-based sitting activities on weekdays, weekend days and over the past 7 days (p<0.001). Moderate to strong positive correlations were observed between the SIT-Q-7d-Sp and the AP^3M^+Log for meal, work and transportation-based sitting time on weekdays, weekend days and for the past 7 days (rho = 0.49–0.67; all p<0.01). No associations were observed between the two measures for any other variables.

**Table 3:** Descriptive characteristics of the average minutes spent in different sedentary domains from the SIT-Q-7d-Sp and the AP^3M^ + Log [mean (standard deviation)], Intraindividual Differences [median (interquartile range)] and results of the Wilcoxon Signed Rank tests.

N = 30	SIT-Q-7d	activPAL + Log	Difference	Rho
**Sitting Time during Meals** **Weekday (mins·d**^**-1**^**)**	68.67 (31.20)	72.56 (27.29)	-3.89 (45.21)	0.50**
**Weekend Day (mins·d**^**-1**^**)**	84.08 (36.29)	84.25 (34.26)	-0.16 (51.03)	0.53**
**Average 7 Day (mins·d**^**-1**^**)**	73.07 (31.36)	76.39 (26.15)	-3.32 (34.69)	0.56**
**Sitting Time during Work** **Weekday (mins·d**^**-1**^**)**	212.80 (146.43)	188.30 (78.23)	7.09 (125.38)	0.49**
**Sitting Time during Transportation Weekday (mins·d**^**-1**^**)**	90.55 (83.52)	70.03 (45.07)	3.30 (75.39)	0.53**
**Weekend Day (mins·d**^**-1**^**)**	61.25 (89.74)	56.28 (44.41)	-10.61 (59.55)	0.49**
**Average 7 Day (mins·d**^**-1**^**)**	82.18 (77.49)	65.50 (40.34)	2.34 (59.11)	0.67***
**Screen-based Leisure Sitting Time** **Weekday (mins·d**^**-1**^**)**	224.75 (146.91)	89.99 (79.93)	121.05 (172.3)***	0.29
**Weekend Day (mins·d**^**-1**^**)**	212.00 (122.13)	102.68 (77.09)	94.68 (128.18)***	0.20
**Average 7 Day (mins·d**^**-1**^**)**	221.11 (124.32)	98.08 (68.07)	113.83 (132.19)***	0.36
**Other Sitting Time** **Weekday (mins·d**^**-1**^**)**	270.00 (191.91)	52.68 (53.87)	184.61 (234.80)***	0.23
**Weekend Day (mins·d**^**-1**^**)**	314.25 (218.88)	63.55 (61.15)	234.09 (403.48)***	-0.12
**Average 7 Day (mins·d**^**-1**^**)**	282.64 (190.86)	56.55 (45.87)	192.69 (263.10)***	0.05

* P<0.016;

** P<0.01;

*** P<0.001

**Fig 3:**
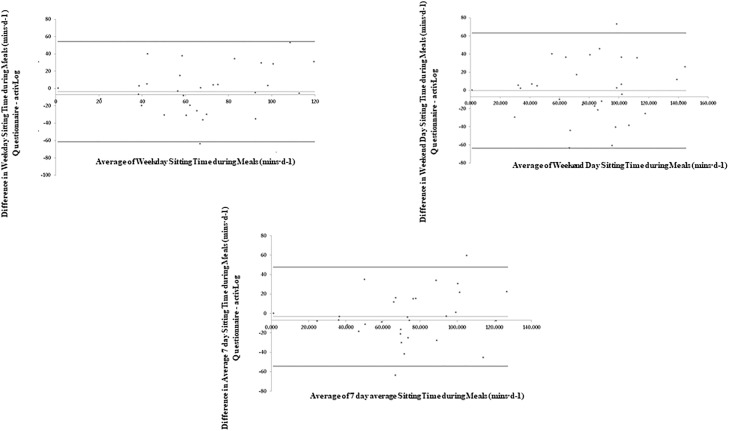
Bland-Altman plots of absolute agreement of sitting time during meal time (weekdays, weekend days and average of last 7 days) derived from the questionnaire with the equivalent variable derived from the criterion measure (combination of AP^3M^ determined sedentary time and the domain specific log).

**Fig 4:**
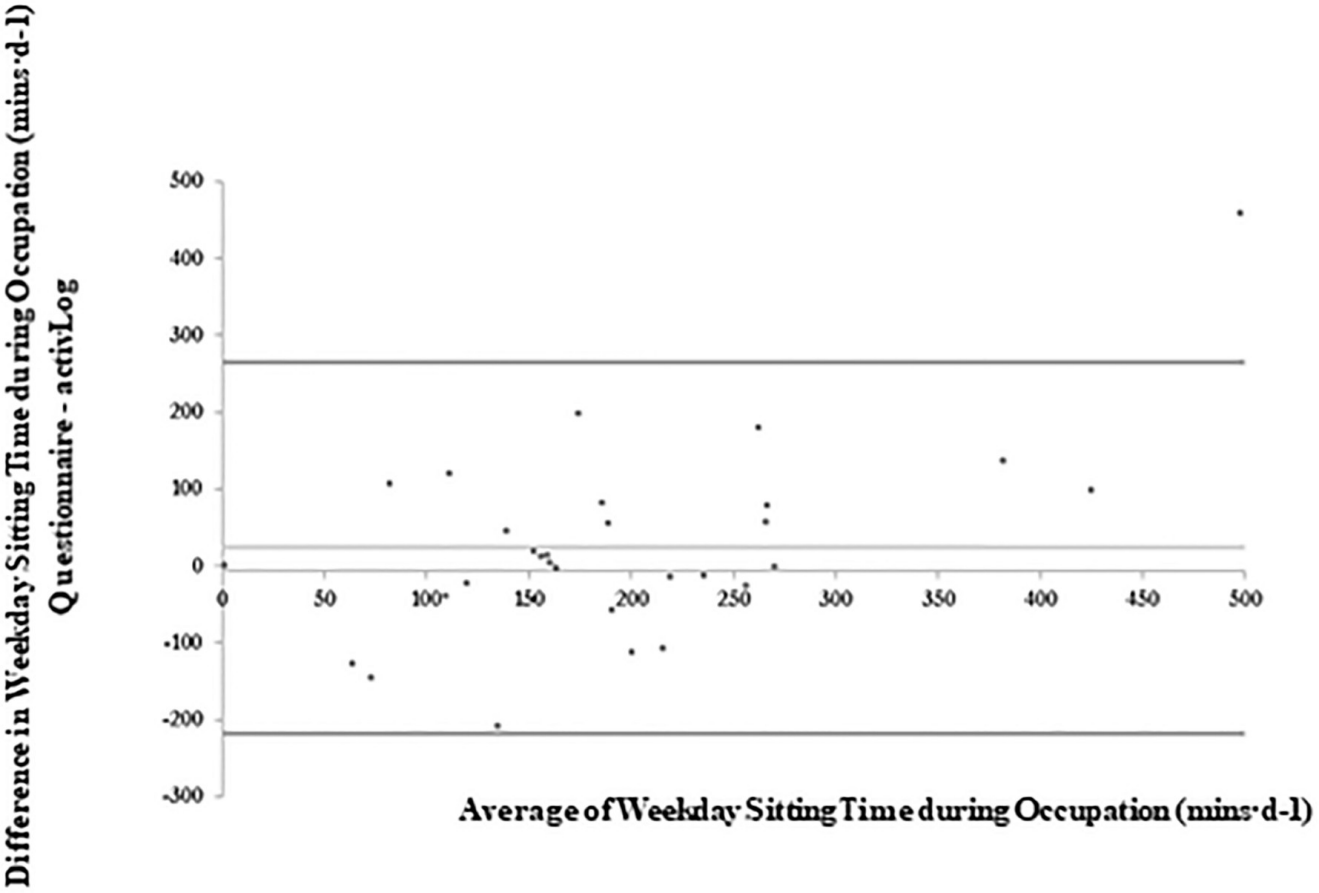
Bland-Altman plots of absolute agreement of sitting time during occupational time (weekdays) derived from the questionnaire with the equivalent variable derived from the criterion measure (combination of AP^3M^ determined sedentary time and the domain specific log).

**Fig 5:**
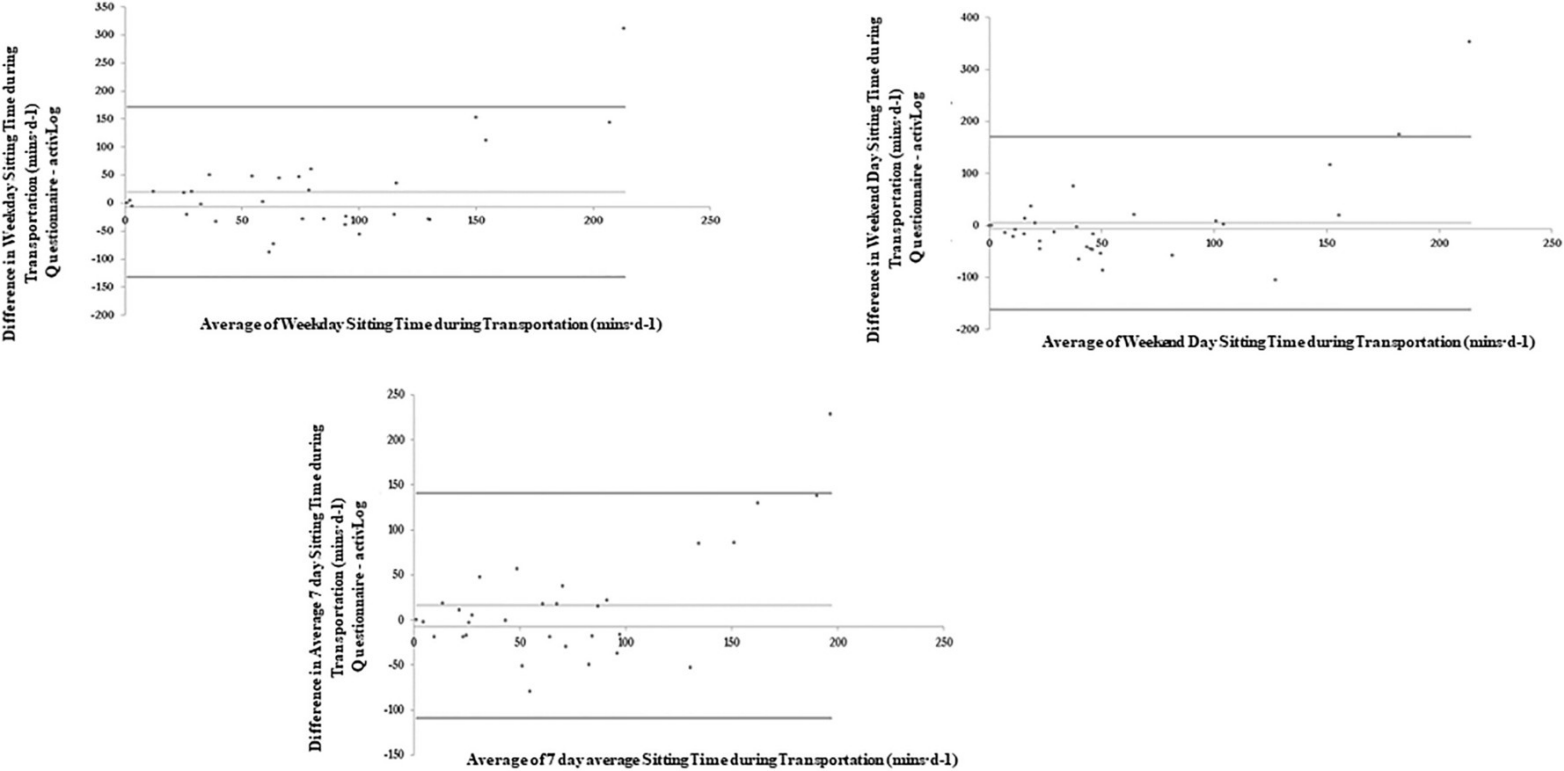
Bland-Altman plots of absolute agreement of sitting time during transportation time (weekdays, weekend days and average of last 7 days) derived from the questionnaire with the equivalent variable derived from the criterion measure (combination of AP^3M^ determined sedentary time and the domain specific log).

**Fig 6:**
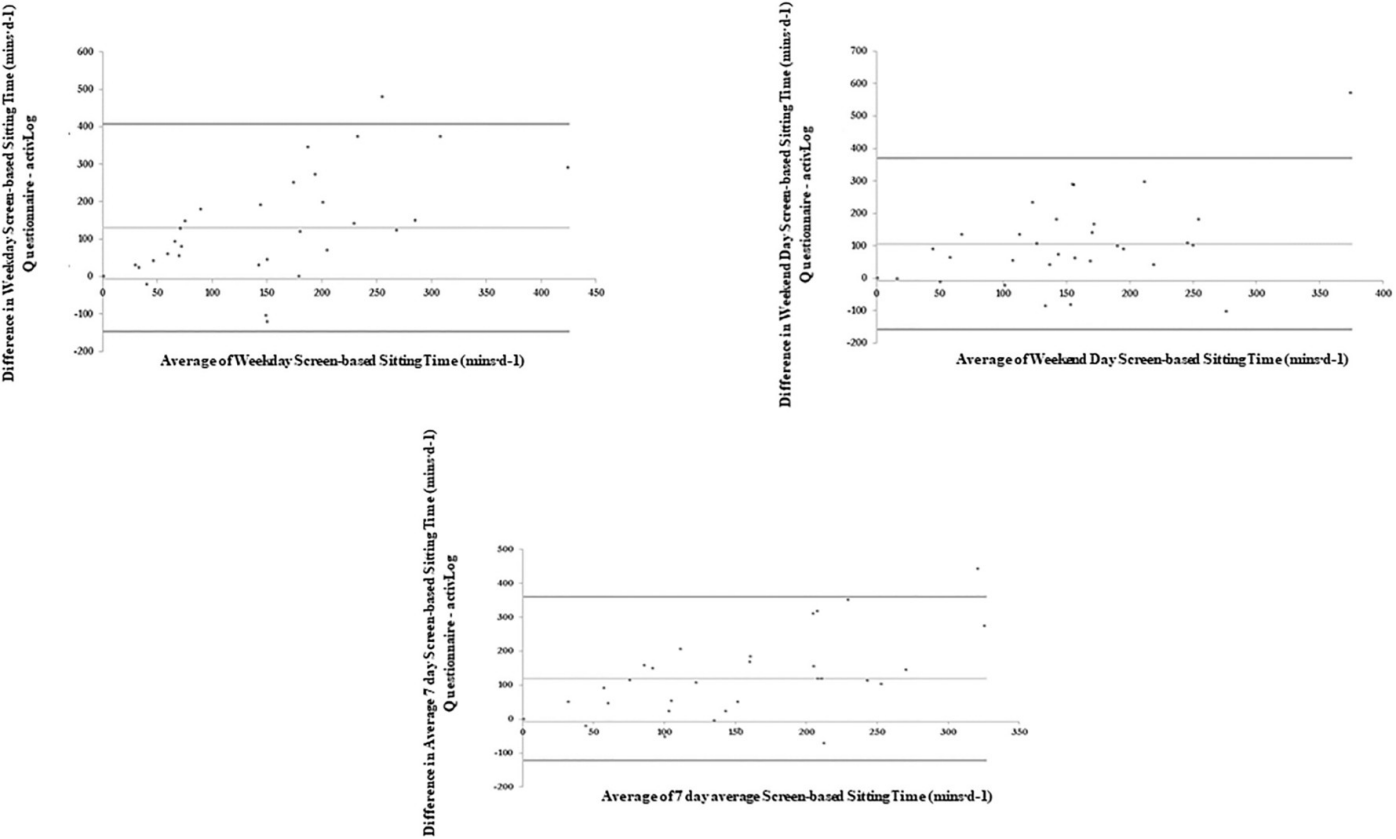
Bland-Altman plots of absolute agreement of screen-based sitting time (weekdays, weekend days and average of last 7 days) derived from the questionnaire with the equivalent variable derived from the criterion measure (combination of AP^3M^ determined sedentary time and the domain specific log).

**Fig 7:**
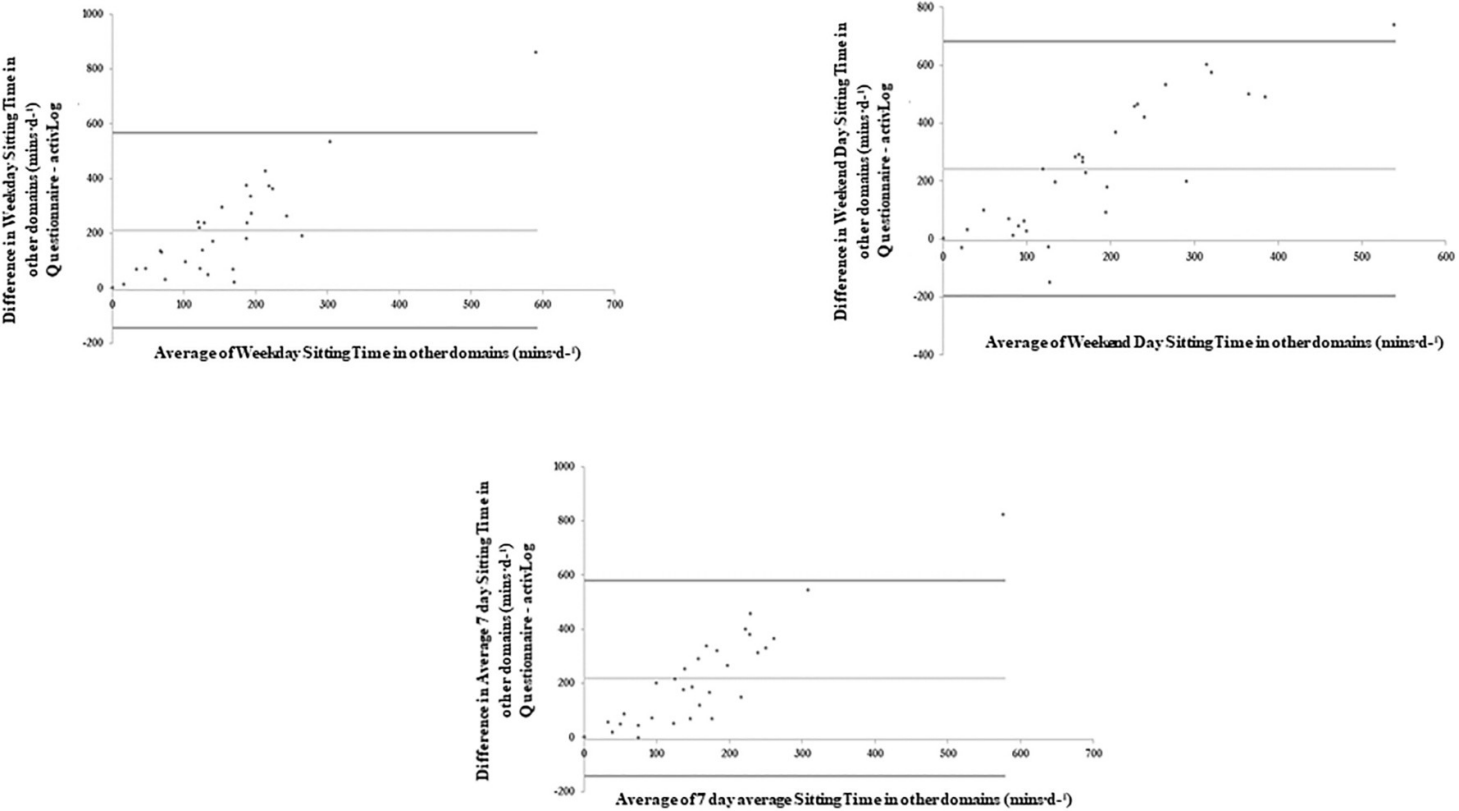
Bland-Altman plots of absolute agreement of sitting time in all other non-screen leisure domains (weekdays, weekend days and average of last 7 days) derived from the questionnaire with the equivalent variable derived from the criterion measure (combination of AP^3M^ determined sedentary time and the domain specific log).

Table 4 and Figs 3–7 present the Bland-Altman plots comparing the estimates of sitting time between the two measures for all domains. The mean bias observed between the SIT-Q-7d-Sp and the AP^3M^ +Log for sitting time during meals, at work and during transportation were relatively small when examined for weekdays, weekend days and over the past 7days. For screen-based sitting time on weekdays, weekend days and on the past 7 days, participant’s significantly over-reported sedentary time compared to the AP^3M^ +Log (130.42 mins·d^-1^, 105.80 mins·d^-1^ and 119.07 mins·d^-1^ respectively). A strong positive correlation was observed in the Bland-Altman plots for leisure screen-based sitting time on weekdays (rho = 0.62; p<0.001), weekend days (rho = 0.62; p<0.001) and over the previous 7 days (rho = 0.62; p<0.001), suggesting that those accumulating higher levels of sitting time over-reported this behaviour to a greater extent than those with lower levels.

**Table 4:** Mean bias and limits of agreement for domain specific sedentary time on weekdays, weekend days and average 7-days.

	Mean Bias	Upper LoA	Lower LoA
**Sitting Time during Meals**			
**Weekday (mins·d**^**-1**^**)**	-3.76	54.12	-61.63
**Weekend Day (mins·d**^**-1**^**)**	-0.15	63.37	-63.67
**Average 7 Day (mins·d**^**-1**^**)**	-3.21	47.66	-54.07
**Sitting Time during Work**			
**Weekday (mins·d**^**-1**^**)**	23.71	264.80	-217.37
**Sitting Time during Transportation**			
**Weekday (mins·d**^**-1**^**)**	19.87	171.90	-132.16
**Weekend Day (mins·d**^**-1**^**)**	4.82	170.99	-161.35
**Average 7 Day (mins·d**^**-1**^**)**	16.14	140.86	-108.57
**Screen-based Sitting Time**			
**Weekday (mins·d**^**-1**^**)**	130.42	407.24	-146.41
**Weekend Day (mins·d**^**-1**^**)**	105.80	369.38	-157.79
**Average 7 Day (mins·d**^**-1**^**)**	119.07	359.86	-121.72
**Other Sitting Time**			
**Weekday (mins·d**^**-1**^**)**	210.31	566.50	-145.87
**Weekend Day (mins·d**^**-1**^**)**	242.62	681.94	-196.70

LoA: Limits of agreement

Participants again significantly over-reported other leisure-based sitting time (includes sitting while reading, performing household tasks, providing care for relatives, performing hobbies, socializing, listening to music) on weekdays, weekend days and for the past 7 days (210.31 mins·d^-1^, 242.62 mins·d^-1^ and 218.81 mins·d^-1^ respectively). A strong positive association (rho = 0.73; p<0.001) was observed between the difference and the average of the SIT-Q-7d-Sp and the AP^3M^ +Log determined other leisure-based sitting time. A very strong positive association was observed between the difference and the average of the SIT-Q-7d-Sp and the AP^3M^ +Log determined other leisure-based sitting time on weekend days (rho = 0.85; p<0.001) and over the past 7 days (rho = 0.83; p<0.001). These findings suggest that those with the highest levels of AP^3M^ +Log determined screen time and other leisure-based sitting time over-reported sitting time to a greater extent than those with lower levels of sitting time.

## Discussion

The purpose of this study was to develop the SIT-Q-7d-Sp through linguistic and cultural adaptation and examine its criterion validity in subsample of the Peninsular-Spanish population. The Spanish version of the SIT-Q-7d-Sp differed significantly from the criterion measure for total sitting time on weekdays and for total sitting time for the past 7 days, overestimating sitting time by 73.46 mins.d^-1^ and by 60.69 mins.d^-1^ respectively. The SIT-Q-7d-Sp did not differ significantly from the AP^3M^ +Log for meal, work and transportation-based sitting time on weekdays, on weekend days and over the past 7 days. However, the SIT-Q-7d-Sp significantly overestimated both leisure screen-based sitting time and other leisure-based sitting activities on all measured days (ranging from 94.68 mins.d^-1^ to 234.08 mins.d^-1^, all p<0.001).

The results of the SIT-Q-7d-Sp highlight differences in sitting time based on all day measurements. Weekday sitting time differed significantly from the criterion measure, yet no significant difference was observed between the SIT-Q-7d-Sp and the AP^3M^ on weekend days. However, there were no significant associations observed between the two measures for weekend sitting time (rho = 0.14; p>0.016). When examined collectively, sitting time for the past 7 days from the SIT-Q-7d-Sp was overestimated when compared to the AP^3M^, while weak positive associations were observed between the two measures (rho = 0.28; p<0.001). These findings align with those from the Dutch and English SIT-Q-7d. The Dutch version showed a significant overestimation of sitting time and moderate positive associations (rho = 0.52; p<0.001) when compared to AP^3M^ -determined sitting time [20]. For the English version, Wijndaele and colleagues [20] observed a significant underestimation in sitting time when compared to accelerometer-determined sedentary time (rho = 0.37; p<0.001) and a significant overestimation when compared to accelerometer+heart rate-determined sedentary time (rho = 0.22; p<0.001). Differences observed here are likely due to the measures employed, whereby the AP^3M^ determines postural position based on thigh acceleration, while the ActiHeart determines sedentary behaviour (defined as an energy expenditure <1.5 METs) based on a combination of trunk acceleration and heart rate signal.

The mean bias for total sitting time assessed by the SIT-Q-7d-Sp was large (60.69 mins.d^-1^). Consistent with previous findings, those with the highest levels of sitting time overestimated their sitting time, while those with lower levels of sitting time underestimated their sitting time [18,20,22,33]. The 95% LoA for the Bland-Altman plot for total sitting time were also relatively wide (-385.45, 506.82 mins.d^-1^). These findings suggest that the SIT-Q-7d-Sp, as with the Dutch version [20], is less suitable for the quantification of total sitting time at the individual level, and hence its use for the examination of the effectiveness of interventions that aim to reduce total sitting time is not recommended. It should also be acknowledged that these findings are not limited to the SIT-Q-7d-Sp, as other self-reported measures of SB [18,33] have identified similar overestimations of total SB, with similar trends across different levels of sedentariness (i.e. underestimation at lower levels and overestimations at higher levels) when compared to objectively determined postural position).

The domain-specific sitting indicators for transportation, work and meal time were significantly correlated with the criterion measure (rho = 0.49–0.67). However, leisure screen-based and other non-screen leisure-based sitting time from the SIT-Q-7d-Sp differed significantly from the criterion measure, with no significant correlations observed for weekdays, weekend days or past 7 days for these domains. These two domains were the most predominant influencers for total sedentary time overestimation, with large discrepancies between the SIT-Q-7d-Sp and the criterion validity at the individual level. For non-screen leisure sitting time, these findings are consistent with other studies, [13,16,20,22,34] whereby poor criterion validity for other leisure-based sitting activities (i.e. sitting while reading, performing household tasks, providing care for relatives, performing hobbies, socializing, listening to music) was observed. This is likely due to the activity types, as these activities are shorter and more sporadic in nature than other sedentary activities that have more structure, such as occupational and commute-based sitting time and time spend sitting during meals.

In contrast to the original version of the SIT-Q-7d [20], and other measures of SB (e.g. MSQ [16]), screen-based sitting time was not accurately self-reported for the SIT-Q-7d-Sp. A possible explanation may be due to the age of participants in our sample (21.19 ±2.57 yrs.) compared to the other validations (mean average >39 yrs.). Young people interact distinctly with screens, with more sporadic leisure screen time usage through computers, tablets and smartphones, while engaging in other tasks such as listening to music or watching TV [23,33]. Due to the omnipresence of screens in the modern daily lives of young people, the traditional use of “screen-time” as a surrogate of sitting time may be problematic, as these populations may now accumulate substantially more time engaged with screens while not in a sitting/lying posture.

### Strengths and limitations

The homogeneity of the sample (young adult students) and the relatively small sub-sample used for the domain-specific validation must be identified as key limitations of this study, which reduce the generalisability of the findings. Another limitation is the higher percentages of males for the domain-specific validation subgroup compared to the total sample (70% vs 52% respectively). Future studies should focus on the criterion validity of the SIT-Q-7d-Sp with other populations across different ages, education levels and occupation types. Additionally, although participants were asked to complete the domain log during the day, they may have done it retrospectively, thus, the measure per se may suffer from the same recall bias as all other self-reported behaviours.

The strengths of this study should be acknowledged. This study employed the “gold standard” objective measure [11] of sitting time as the comparison for the SIT-Q-7d-Sp measure. Additionally, the use of a domain-log to determine domain specific sitting time from the AP^3M^ enabled more specific comparisons between the SIT-Q-7d-Sp and the objective measure. This is the first study that adapted, translated and validated the SIT-Q 7d for Spanish-speaking population with the objective “gold standard” measure of sitting behaviour. The findings of this study contribute to the scarcely available information on validated self-administered SB instruments for the general Spanish-speaking population. Having language-specific questionnaires may improve our understanding of prevalence of SB worldwide and its associations with health and its determinants in different settings.

## Conclusions

The SIT-Q-7d-Sp provides acceptable estimates of sitting time in the transportation, occupational and meal-based domains. However, it significantly overestimates total sitting time, screen-based sitting time and other leisure-based sitting activities. The findings of this study suggest that the SIT-Q-7d-Sp is not an appropriate measure of total sitting time and leisure-based sedentary activities in young adults. This study contributes to the rationale of the measurement-based evidence supporting the lack of accuracy of self-reported instruments to measure total sitting time and unstructured domain-specific sitting time, as well as the ability of such measures to detect behaviour changes. For SIT-Q-7d-Sp derived total sitting time, it is recommended to compute variables over the past 7 days rather than for total sitting time on weekdays and weekend days. In addition, for total sitting time and leisure-based sitting time, the use of objective measures is recommended.

## Supporting information

S1 Appendix Spanish version of the last 7 days SB questionnaire (SIT-Q-7d-Sp). (PDF)

S2 Appendix Spanish domain-log. (PDF)

S3 Appendix SIT-Q-7d-Sp validation study data. (XLSX)
