# Supplementary material for: Validation study of the Spanish version of the Last-7-d Sedentary Time Questionnaire (SIT-Q-7d-Sp) in young adults
Source: PLoS One. 2019 May 29;14(5):e0217362. doi: 10.1371/journal.pone.0217362 (PMC6541286; doi:10.1371/journal.pone.0217362)
Supplement: S1 Appendix — (PDF) [file pone.0217362.s001.pdf]

SIT-Q-7d-S

Se le formularán preguntas acerca de la cantidad de tiempo que ha ***pasado sentado/a o tumbado/a*** *en los* ***últimos 7 días.***

1. *Introduzca cada periodo que ha pasado sentado/a* ***una sola vez*** *en el cuestionario. Por ejemplo, si estuvo sentado/a en el sofá durante una hora leyendo y escuchando música a la vez, este periodo contará como una hora de lectura si esa era la tarea principal. No lo cuente como una hora escuchando música también.*
2. *Si hay una diferencia notable entre días laborables o entre días no laborables, marque la casilla que más se acerca a la* ***media*** *de esos días laborables o no laborables* ***en los últimos 7 días****.*
3. *Este cuestionario se centra en los momentos que ha pasado* ***sentado/a o tumbado/a*** *mientras realizaba las actividades que se mencionan a continuación. Si durante la realización de estas actividades también estuvo de pie o caminando, intente incluir solamente el tiempo que pasó sentado/a o tumbado/a durante la actividad.*

1. Compare la cantidad de tiempo que ha pasado sentado/a en los **últimos 7 días** con respecto a una **semana normal para usted**. En los últimos 7 días, la cantidad de tiempo que he pasado sentado/da ha sido….

Por favor marque ***sólo*** *una casilla*

Mucho menos de lo normal	Un poco menos de lo normal	Más o menos igual	Un poco más de lo normal	Mucho más de lo normal
☐	☐	☐	☐	☐

A. DORMIR Y SIESTA

Piense en la hora a la que se fue a dormir y se levantó en los últimos 7 días. Si tuvo patrones de sueño cambiantes (ej. cambios de turnos de trabajo), calcule la hora media a la que se fue a dormir y se levantó los días laborables y no laborables.

➔ NO INCLUYA:

- *El tiempo que ha pasado leyendo o viendo la TV antes de dormir o después de despertarse.*

2. En los últimos 7 días, ¿a qué hora se acostó y se levantó?

	Hora de acostarse	Hora de levantarse
**Día laborable**	______ :______	______ :______
**Día no laborable**	______ :______	______ :______

3. En los últimos 7 días, de media, ¿cuánto tiempo de siesta hizo al día?

Por favor marque ***sólo*** *una casilla por línea*

	0	1-15 min	15-30 min	30-45 min	45 min-1 hora	Más de 1 hora al día
**Día laborable**	☐	☐	☐	☐	☐	☐
**Día no laborable**	☐	☐	☐	☐	☐	☐

B. COMIDAS

Piense en la cantidad de tiempo de media que estuvo sentado/a durante el desayuno, almuerzo y cena en los últimos 7 días

➔ INCLUYA:

- *el tiempo que ha pasado sentado/a durante el desayuno, el almuerzo y la cena (en casa, en el trabajo…) mientras leía, hablaba con otras personas o escuchaba la radio. Por ejemplo, si estuvo sentado/a 30 minutos durante el desayuno mientras leía el periódico o durante el almuerzo mientras trabajaba inclúyalo en este apartado.*

➔ NO INCLUYA:

- *tiempo pasado comiendo mientras veía la TV. Esto será incluido más adelante.*

4. En los últimos 7 días, de media al día ¿cuánto tiempo estuvo sentado/a durante cada una de las comidas?

Por favor marque ***sólo*** *una casilla por línea*

		0	1-10 min	10-20 min	20-30 min	30-45 min	45 min-1 hora	Más de 1 hora al día
**Día laborable**	Desayuno	☐	☐	☐	☐	☐	☐	☐
	Almuerzo	☐	☐	☐	☐	☐	☐	☐
	Cena	☐	☐	☐	☐	☐	☐	☐
**Día no laborable**	Desayuno	☐	☐	☐	☐	☐	☐	☐
	Almuerzo	☐	☐	☐	☐	☐	☐	☐
	Cena	☐	☐	☐	☐	☐	☐	☐

C. DESPLAZAMIENTOS

Este apartado se centra en la cantidad de tiempo que pasó sentado/a mientras realizaba desplazamientos (en un coche, en un autobús, en un tren, en una moto…) en los últimos 7 días. Se le formularán preguntas acerca de los desplazamientos hacia y desde su ocupación, los desplazamientos como parte de su ocupación y los desplazamientos que no forman parte de su ocupación.

"Ocupación" hace referencia a tres tipos de actividades diferentes: trabajo, estudio y voluntariado. "Trabajo" consiste en todas las tareas realizadas con remuneración. "Estudio" se refiere a actividades educativas y formativas. "Voluntariado" hace referencia al trabajo que realiza sin remuneración, como ayudar en un club deportivo. Piense en estas tres categorías para responder a las siguientes preguntas.

➔ NO INCLUYA:

- *ir en una bicicleta de pedales*

5. ¿Ha trabajado, estudiado o realizado un voluntariado (entendido como una "ocupación" en los últimos 7 días?

☐ Sí ☐ No (En este caso vaya al apartado 3D DESPLAZAMIENTOS APARTE DE SU OCUPACIÓN)

6. En los últimos 7 días, ¿cuántos días a la semana se sentó usted mientras se desplazaba hacia y desde su ocupación? (en un coche, autobús, tren, moto, etc.; no incluya ir en una bicicleta de pedales).

En caso que sea necesario, dispone de espacio para dos ocupaciones diferentes.

Ocupación 1: _______días Ocupación 2: ______ días

7. En los últimos 7 días, de media, ¿cuánto tiempo pasó usted sentado/a mientras se desplazaba hacia y desde su ocupación en un día cualquiera? (en un coche, autobús, tren, moto, etc.; no incluya ir en una bicicleta de pedales)

*Por favor marque **sólo** una casilla por línea*

	0	1-15 min	15-30 min	30-45 min	45 min-1 hora	1-1,5 horas	1,5-2 horas	2-2,5 horas	2,5-3 horas	3-4 horas	4-5 horas	5-6 horas	6-7 horas	Más de 7 horas
**Ocupación 1**	☐	☐	☐	☐	☐	☐	☐	☐	☐	☐	☐	☐	☐	☐
**Ocupación 2**	☐	☐	☐	☐	☐	☐	☐	☐	☐	☐	☐	☐	☐	☐

D. DESPLAZAMIENTOS APARTE DE SU OCUPACIÓN

Piense en el tiempo que pasa sentado/a mientras realiza desplazamientos aparte de su ocupación, por ejemplo, yendo al supermercado, a visitar amigos, etc. Por favor incluya el tiempo que ha pasado sentado/a hacia y desde su destino.

8. En los últimos 7 días, de media al día, ¿cuánto tiempo estuvo usted sentado/a en los desplazamientos aparte de su ocupación? (en un coche, autobús, tren, moto, etc.; no incluya ir en una bicicleta de pedales)

*Por favor marque **sólo** una casilla por línea*

	0	1-15 min	15-30 min	30-45 min	45 min- 1 hora	1-1,5 horas	1,5-2 horas	2-2,5 horas	2,5-3 horas	3-4 horas	4-5 horas	5-6 horas	6-7 horas	Más de 7 horas
**Día laborable**	☐	☐	☐	☐	☐	☐	☐	☐	☐	☐	☐	☐	☐	☐
**Día no laborable**	☐	☐	☐	☐	☐	☐	☐	☐	☐	☐	☐	☐	☐	☐

E. TRABAJO, ESTUDIO Y VOLUNTARIADO

Este apartado trata del tiempo que usted pasa sentado/a durante su ocupación, entendido como trabajo, estudio y voluntariado. Por favor, piense en estas tres categorías para contestar a las siguientes preguntas.

9. ¿Tuvo usted una "ocupación" en los últimos 7 días (trabajo, estudio o voluntariado)?

☐ No (En este caso vaya al apartado 3F TIEMPO DELANTE DE UNA PANTALLA)

☐ Sí (En este caso complete el apartado a continuación. Dispone de espacio para dos ocupaciones diferentes).

10. **Ocupación 1**. Tipo de ocupación 1

☐ Trabajo ☐ Estudio ☐ Voluntariado

11. Nombre de la ocupación (ej. estudiante, recepcionista…)

____

12. ¿Cuántos días realizó la ocupación 1 en los últimos 7 días?

_____________ días

13. En los últimos 7 días, de media ¿cuánto tiempo al día pasó sentado/a mientras realizaba la ocupación 1?

*Por favor marque **sólo** una casilla por línea*

➔ NO INCLUYA:

• tiempo sentado/a durante un desplazamiento (en un coche, autobús, tren, moto, etc.) ya sea para ir o para volver de su ocupación, o como parte de ella.

• desayuno, almuerzo o cena.

0	1-15 min	15-30 min	30min-1hora	1-2 horas	2-3 horas	3-4 horas	4-5 horas	5-6 horas	6-7 horas	7-8 horas	Más de 8 horas
☐	☐	☐	☐	☐	☐	☐	☐	☐	☐	☐	☐

14. ¿Tuvo usted una segunda ocupación en los últimos 7 días?

☐ Sí

☐ No (En este caso vaya al apartado 3F TIEMPO DELANTE DE UNA PANTALLA)

15. **Ocupación 2.** Tipo de ocupación 2

☐ Trabajo ☐ Estudio ☐ Voluntariado

16. Nombre de la ocupación (ej. estudiante, recepcionista…)

____

17. ¿Cuántos días realizó la ocupación 2 en los últimos 7 días?

____________ días

18. En los últimos 7 días, de media ¿cuánto tiempo al día pasó sentado/a mientras realizaba la ocupación 2?

*Por favor marque **sólo** una casilla por línea*

➔ NO INCLUYA:

• tiempo sentado/a durante un desplazamiento (en un coche, autobús, tren, moto, etc.) ya sea para ir o para volver de su ocupación, o como parte de ella.

• desayuno, almuerzo o cena.

0	1-15 min	15-30 min	30min-1hora	1-2 horas	2-3 horas	3-4 horas	4-5 horas	5-6 horas	6-7 horas	7-8 horas	Más de 8 horas
☐	☐	☐	☐	☐	☐	☐	☐	☐	☐	☐	☐

F. TIEMPO DELANTE DE UNA PANTALLA

Este apartado hace referencia al tiempo que estuvo sentado/a o tumbado/a mientras realizaba otras actividades en los últimos 7 días. Recuerde que debe introducir cada periodo que ha pasado sentado/a una sola vez. Por ejemplo, si estuvo sentado/a en el sofá durante una hora leyendo y escuchando música a la vez, este periodo contará como una hora de lectura si esa era la tarea principal. No lo cuente como una hora escuchando música también.

19. En últimos 7 días, de media ¿cuánto tiempo al día permaneció sentado/a o tumbado/a mientras realizaba las siguientes actividades?

*Por favor marque **sólo** una casilla por línea*

		0	1-15 min	15-30 min	30 min- 1h	1-2 h	2-3 h	3-4 h	4-5 h	5-6 h	6-7 h	Más de 7h
**Viendo la TV, DVD/vídeos**	**Día laborable**	☐	☐	☐	☐	☐	☐	☐	☐	☐	☐	☐
**➔ INCLUYA** comidas sentado/a mientras ve la TV	**Día no laborable**	☐	☐	☐	☐	☐	☐	☐	☐	☐	☐	☐
**Usando el ordenador/tablet o smartphone fuera del trabajo** *(internet, correo*	**Día laborable**	☐	☐	☐	☐	☐	☐	☐	☐	☐	☐	☐
electrónico, chat, redes sociales (Facebook,…)	**Día no laborable**	☐	☐	☐	☐	☐	☐	☐	☐	☐	☐	☐
**Jugando a videojuegos** *(Playstation, Xbox,…)*	**Día laborable**	☐	☐	☐	☐	☐	☐	☐	☐	☐	☐	☐
**➔ NO INCLUYA** los que no se jueguen sentado	**Día no laborable**	☐	☐	☐	☐	☐	☐	☐	☐	☐	☐	☐

G. OTRAS ACTIVIDADES

Por favor recuerde que debe introducir cada periodo que ha pasado sentado/a una sola vez.

20. En los últimos 7 días, de media ¿cuánto tiempo al día permaneció sentado/a mientras realizaba las siguientes actividades?

*Por favor marque **sólo** una casilla por línea*

		0	1-15 min	15-30 min	30 min- 1 hora	1-2 horas	2-3 horas	3-4 horas	4-5 horas	5-6 horas	6-7 horas	Más de 7 horas
**Sentado/a mientras lee o estudia** (libro, revistas, periódico…)	**Día laborable**	☐	☐	☐	☐	☐	☐	☐	☐	☐	☐	☐
	**Día no laborable**	☐	☐	☐	☐	☐	☐	☐	☐	☐	☐	☐
**Sentado/a mientras realiza tareas del hogar** (cocinar, planchar…)	**Día laborable**	☐	☐	☐	☐	☐	☐	☐	☐	☐	☐	☐
	**Día no laborable**	☐	☐	☐	☐	☐	☐	☐	☐	☐	☐	☐
**Sentado/a mientras cuida** niños, familiares ancianos o inválidos…	**Día laborable**	☐	☐	☐	☐	☐	☐	☐	☐	☐	☐	☐
	**Día no laborable**	☐	☐	☐	☐	☐	☐	☐	☐	☐	☐	☐
**Sentado/a mientras realiza pasatiempos** (piano, cartas, crucigramas…)	**Día laborable**	☐	☐	☐	☐	☐	☐	☐	☐	☐	☐	☐
	**Día no laborable**	☐	☐	☐	☐	☐	☐	☐	☐	☐	☐	☐
**Sentado/a mientras socializa** (visitar amigos, en el bar, el cine, eventos deportivos…)	**Día laborable**	☐	☐	☐	☐	☐	☐	☐	☐	☐	☐	☐
	**Día no laborable**	☐	☐	☐	☐	☐	☐	☐	☐	☐	☐	☐
**Sentado/a mientras escucha música** (radio, CD,MP3, iPOD…)	**Día laborable**	☐	☐	☐	☐	☐	☐	☐	☐	☐	☐	☐
	**Día no laborable**	☐	☐	☐	☐	☐	☐	☐	☐	☐	☐	☐
**Sentado/a durante otras actividades** (especifique aquí):	**Día laborable**	☐	☐	☐	☐	☐	☐	☐	☐	☐	☐	☐
	**Día no laborable**	☐	☐	☐	☐	☐	☐	☐	☐	☐	☐	☐

Ha finalizado el cuestionario.

Muchas gracias por su colaboración.

Cuestionario original en Inglés desarrollado y validado por:
Wijndaele, K., et al. (2014). Reliability and validity of a domain-specific last 7-d sedentary time questionnaire. *Medicine and Science in Sports and Exercise*, *46*(6), 1248–1260.

Adaptación y validación al Español:
Felez-Nobrega et al., (2018). Validation study of the Spanish version of the Last-7-d Sedentary Time Questionnaire. *PLOS ONE*
