# Supplementary material for: Validation study of the Spanish version of the Last-7-d Sedentary Time Questionnaire (SIT-Q-7d-Sp) in young adults
Source: PLoS One. 2019 May 29;14(5):e0217362. doi: 10.1371/journal.pone.0217362 (PMC6541286; doi:10.1371/journal.pone.0217362)
Supplement: S2 Appendix — (PDF) [file pone.0217362.s002.pdf]

DIARIO DE REGISTRO

Si tiene alguna duda póngase

en contacto con el equipo investigador:

¡Muchas gracias por su colaboración!

UVIC

¿Cómo relleno este registro?

- Este registro está dividido en 8 días (del día 0 al día 7). Una hoja por día. Rellénelo durante todo el período en que está usando el acelerómetro. Intente siempre llevar el registro consigo, así podrá rellenarlo durante el transcurso del día. Esto asegurará que la información que proporcione es exacta y que no tiene que confiar en su memoria. En la página siguiente hay un ejemplo de cómo llenar el registro.

- Cada tabla muestra las 24 horas de ese día (filas), y todas las horas del día se dividen en cuatro períodos de 15 minutos (columnas). En el inicio de la jornada, la medianoche está representada por el cuadro de arriba a la izquierda: "Hora 0, Min 0-15".

- Escriba en cada casilla el número que corresponda a la categoría de actividades que se realizan durante ese período de 15 minutos.

- Si realiza una actividad por un período de más de 15 minutos (por ejemplo dormir), es posible trazar una línea horizontal a través de los cuadros siguientes, hasta el cuadro donde cambia la categoría de la actividad. Si realiza dos actividades simultáneamente rellene ambas cifras (por ej. desayuno durante el desplazamiento en tren, puede rellenar "2/3").

- Si no puede llevar el acelerómetro durante un corto período de tiempo (¡pero sólo cuando sea realmente necesario!), indíquelo en el registro con una "X".

DIA 1 (EJEMPLO)

Fecha:

Instrucciones:

- Escriba en **cada** casilla el número que corresponde a la categoría de actividades de que realiza durante ese período de 15 minutos

Categorías:

- **Incluya únicamente actividades que realice sentado / tumbado**

1. **Duermo/hacer siesta**
2. **Desayuno, como o ceno**
3. **Me desplazo** (en coche, bus, tren, etc.) no importa dónde (trabajo, gimnasio, supermercado, etc.)
4. **Trabajo** (incluyendo todas las tareas que realice para ganar dinero)
5. **Estudio** (asistir a clase y actividades de estudio)
6. **Hacer voluntariado** (trabajo para el que no cobra)
7. **Miro la TV/DVD/vídeos/ smartphone**
8. **Utilizo el ordenador** (fuera del horario lectivo)
9. **Sentado haciendo otras actividades** (socializarse, leer, escuchar música, etc.)

X. **NO** usé el acelerómetro (sólo quítelo cuando sea absolutamente necesario)

Min / hora	0-15	16-30	31-45	46-60
0	1			
1				
2				
3				
4				
5				
6				
7	9			2
8	3			
9	4			
10				
11				
12			2	
13			4	
14				
15				
16				
17			3	
18	9			
19			2	
20	7			8
21				
22	x	1		
23				

DIA 1   Fecha: /   /

Instrucciones:

- Escriba en **cada** casilla el número que corresponde a la categoría de actividades de que realiza durante ese período de 15 minutos

Categorías:

- **Incluya únicamente actividades que realice sentado / tumbado**

1. **Duermo/hacer siesta**
2. **Desayuno, como o ceno**
3. **Me desplazo** (en coche, bus, tren, etc.) no importa dónde (trabajo, gimnasio, supermercado, etc.)
4. **Trabajo** (incluyendo todas las tareas que realice para ganar dinero)
5. **Estudio** (asistir a clase y actividades de estudio)
6. **Hacer voluntariado** (trabajo para el que no cobra)
7. **Miro la TV/DVD/vídeos/ smartphone**
8. **Utilizo el ordenador** (fuera del horario lectivo)
9. **Sentado haciendo otras actividades** (socializarse, leer, escuchar música, etc.)

X. **NO** usé el acelerómetro (sólo quítelo cuando sea absolutamente necesario)

Min / hora	0-15	16-30	31-45	46-60
0				
1				
2				
3				
4				
5				
6				
7				
8				
9				
10				
11				
12				
13				
14				
15				
16				
17				
18				
19				
20				
21				
22				
23				

**DIA 2**	Fecha: / /

Instrucciones:

- Escriba en **cada** casilla el número que corresponde a la categoría de actividades de que realiza durante ese período de 15 minutos

Categorías:

- **Incluya únicamente actividades que realice sentado / tumbado**

1. **Duermo/hacer siesta**
2. **Desayuno, como o ceno**
3. **Me desplazo** (en coche, bus, tren, etc.) no importa dónde (trabajo, gimnasio, supermercado, etc.)
4. **Trabajo** (incluyendo todas las tareas que realice para ganar dinero)
5. **Estudio** (asistir a clase y actividades de estudio)
6. **Hacer voluntariado** (trabajo para el que no cobra)
7. **Miro la TV/DVD/vídeos/ smartphone**
8. **Utilizo el ordenador** (fuera del horario lectivo)
9. **Sentado haciendo otras actividades** (socializarse, leer, escuchar música, etc.)

X. NO usé el acelerómetro (sólo quítelo cuando sea absolutamente necesario)

Min / hora	0-15	16-30	31-45	46-60
0				
1				
2				
3				
4				
5				
6				
7				
8				
9				
10				
11				
12				
13				
14				
15				
16				
17				
18				
19				
20				
21				
22				
23				

DIA 3 Fecha: / /

Instrucciones:

- Escriba en **cada** casilla el número que corresponde a la categoría de actividades de que realiza durante ese período de 15 minutos

Categorías:

- **Incluya únicamente actividades que realice sentado / tumbado**

1. **Duermo/hacer siesta**
2. **Desayuno, como o ceno**
3. **Me desplazo** (en coche, bus, tren, etc.) no importa dónde (trabajo, gimnasio, supermercado, etc.)
4. **Trabajo** (incluyendo todas las tareas que realice para ganar dinero)
5. **Estudio** (asistir a clase y actividades de estudio)
6. **Hacer voluntariado** (trabajo para el que no cobra)
7. **Miro la TV/DVD/vídeos/ smartphone**
8. **Utilizo el ordenador** (fuera del horario lectivo)
9. **Sentado haciendo otras actividades** (socializarse, leer, escuchar música, etc.)

X. **NO** usé el acelerómetro (sólo quítelo cuando sea absolutamente necesario)

Min / hora	0-15	16-30	31-45	46-60
0				
1				
2				
3				
4				
5				
6				
7				
8				
9				
10				
11				
12				
13				
14				
15				
16				
17				
18				
19				
20				
21				
22				
23				

DIA 4

Fecha: / /

Instrucciones:

- Escriba en **cada** casilla el número que corresponde a la categoría de actividades de que realiza durante ese período de 15 minutos

Categorías:

- **Incluya únicamente actividades que realice sentado / tumbado**

1. **Duermo/hacer siesta**
2. **Desayuno, como o ceno**
3. **Me desplazo** (en coche, bus, tren, etc.) no importa dónde (trabajo, gimnasio, supermercado, etc.)
4. **Trabajo** (incluyendo todas las tareas que realice para ganar dinero)
5. **Estudio** (asistir a clase y actividades de estudio)
6. **Hacer voluntariado** (trabajo para el que no cobra)
7. **Miro la TV/DVD/vídeos/ smartphone**
8. **Utilizo el ordenador** (fuera del horario lectivo)
9. **Sentado haciendo otras actividades** (socializarse, leer, escuchar música, etc.)

X. **NO** usé el acelerómetro (sólo quítelo cuando sea absolutamente necesario)

Min / hora	0-15	16-30	31-45	46-60
0				
1				
2				
3				
4				
5				
6				
7				
8				
9				
10				
11				
12				
13				
14				
15				
16				
17				
18				
19				
20				
21				
22				
23				

DIA 5 Fecha: / /

Instrucciones:

- Escriba en **cada** casilla el número que corresponde a la categoría de actividades de que realiza durante ese período de 15 minutos

Categorías:

- **Incluya únicamente actividades que realice sentado / tumbado**

1. **Duermo/hacer siesta**
2. **Desayuno, como o ceno**
3. **Me desplazo** (en coche, bus, tren, etc.) no importa dónde (trabajo, gimnasio, supermercado, etc.)
4. **Trabajo** (incluyendo todas las tareas que realice para ganar dinero)
5. **Estudio** (asistir a clase y actividades de estudio)
6. **Hacer voluntariado** (trabajo para el que no cobra)
7. **Miro la TV/DVD/vídeos/ smartphone**
8. **Utilizo el ordenador** (fuera del horario lectivo)
9. **Sentado haciendo otras actividades** (socializarse, leer, escuchar música, etc.)

X. **NO** usé el acelerómetro (sólo quítelo cuando sea absolutamente necesario)

Min / hora	0-15	16-30	31-45	46-60
0				
1				
2				
3				
4				
5				
6				
7				
8				
9				
10				
11				
12				
13				
14				
15				
16				
17				
18				
19				
20				
21				
22				
23				

DIA 6

Fecha: / /

Instrucciones:

- Escriba en **cada** casilla el número que corresponde a la categoría de actividades de que realiza durante ese período de 15 minutos

Categorías:

- **Incluya únicamente actividades que realice sentado / tumbado**

1. **Duermo/hacer siesta**
2. **Desayuno, como o ceno**
3. **Me desplazo** (en coche, bus, tren, etc.) no importa dónde (trabajo, gimnasio, supermercado, etc.)
4. **Trabajo** (incluyendo todas las tareas que realice para ganar dinero)
5. **Estudio** (asistir a clase y actividades de estudio)
6. **Hacer voluntariado** (trabajo para el que no cobra)
7. **Miro la TV/DVD/vídeos/ smartphone**
8. **Utilizo el ordenador** (fuera del horario lectivo)
9. **Sentado haciendo otras actividades** (socializarse, leer, escuchar música, etc.)

X. NO usé el acelerómetro (sólo quítelo cuando sea absolutamente necesario)

Min / hora	0-15	16-30	31-45	46-60
0				
1				
2				
3				
4				
5				
6				
7				
8				
9				
10				
11				
12				
13				
14				
15				
16				
17				
18				
19				
20				
21				
22				
23				

DIA 7 Fecha: / /

Instrucciones:

- Escriba en **cada** casilla el número que corresponde a la categoría de actividades de que realiza durante ese período de 15 minutos

Categorías:

- **Incluya únicamente actividades que realice sentado / tumbado**

1. **Duermo/hacer siesta**
2. **Desayuno, como o ceno**
3. **Me desplazo** (en coche, bus, tren, etc.) no importa dónde (trabajo, gimnasio, supermercado, etc.)
4. **Trabajo** (incluyendo todas las tareas que realice para ganar dinero)
5. **Estudio** (asistir a clase y actividades de estudio)
6. **Hacer voluntariado** (trabajo para el que no cobra)
7. **Miro la TV/DVD/vídeos/ smartphone**
8. **Utilizo el ordenador** (fuera del horario lectivo)
9. **Sentado haciendo otras actividades** (socializarse, leer, escuchar música, etc.)

X. **NO** usé el acelerómetro (sólo quítelo cuando sea absolutamente necesario)

Min / hora	0-15	16-30	31-45	46-60
0				
1				
2				
3				
4				
5				
6				
7				
8				
9				
10				
11				
12				
13				
14				
15				
16				
17				
18				
19				
20				
21				
22				
23				
